# MethPat: a tool for the analysis and visualisation of complex methylation patterns obtained by massively parallel sequencing

**DOI:** 10.1186/s12859-016-0950-8

**Published:** 2016-02-24

**Authors:** Nicholas C. Wong, Bernard J. Pope, Ida L. Candiloro, Darren Korbie, Matt Trau, Stephen Q. Wong, Thomas Mikeska, Xinmin Zhang, Mark Pitman, Stefanie Eggers, Stephen R. Doyle, Alexander Dobrovic

**Affiliations:** Translational Genomics and Epigenomics Laboratory, Olivia Newton-John Cancer Research Institute, Heidelberg, Victoria 3084 Australia; Murdoch Childrens Research Institute, The Royal Children’s Hospital, Parkville, Victoria 3052 Australia; Department of Paediatrics, The University of Melbourne, Parkville, Victoria 3052 Australia; Victorian Life Sciences Computation Initiative (VLSCI), The University of Melbourne, Parkville, Victoria 3052 Australia; Department of Computing and Information Systems, The University of Melbourne, Parkville, Victoria 3052 Australia; Department of Pathology, The University of Melbourne, Parkville, Victoria 3010 Australia; Centre for Personalised NanoMedicine, Australian Institute of Nanotechnology and Bioengineering, The University of Queensland, Brisbane, Queensland 4072 Australia; School of Chemistry and Molecular Biosciences, University of Queensland, Brisbane, Queensland 4072 Australia; Molecular Pathology Research and Development Laboratory, Department of Pathology, Peter MacCallum Cancer Centre, East Melbourne, Victoria 3002 Australia; BioInfoRx Inc., Madison, WI USA; BioResearch Software Consultants, Battle Ground, WA USA; Department of Animal, Plant and Soil Sciences, La Trobe University, Bundoora, Victoria 3086 Australia; School of Cancer Medicine, La Trobe University, Bundoora, Victoria 3084 Australia; Present Address: Pacific Edge Biotechnology Ltd, Dunedin, Otago 9016 New Zealand; Present Address: Translational Research Laboratory, Division of Cancer Research, Peter MacCallum Cancer Centre, East Melbourne, Victoria 3002 Australia

**Keywords:** DNA methylation, software, visualization, bisulfite, targeted amplicon, epigenetics, epiallele

## Abstract

**Background:**

DNA methylation at a gene promoter region has the potential to regulate gene transcription. Patterns of methylation over multiple CpG sites in a region are often complex and cell type specific, with the region showing multiple allelic patterns in a sample. This complexity is commonly obscured when DNA methylation data is summarised as an average percentage value for each CpG site (or aggregated across CpG sites). True representation of methylation patterns can only be fully characterised by clonal analysis. Deep sequencing provides the ability to investigate clonal DNA methylation patterns in unprecedented detail and scale, enabling the proper characterisation of the heterogeneity of methylation patterns. However, the sheer amount and complexity of sequencing data requires new synoptic approaches to visualise the distribution of allelic patterns.

**Results:**

We have developed a new analysis and visualisation software tool “Methpat”, that extracts and displays clonal DNA methylation patterns from massively parallel sequencing data aligned using Bismark. Methpat was used to analyse multiplex bisulfite amplicon sequencing on a range of CpG island targets across a panel of human cell lines and primary tissues. Methpat was able to represent the clonal diversity of epialleles analysed at specific gene promoter regions. We also used Methpat to describe epiallelic DNA methylation within the mitochondrial genome.

**Conclusions:**

Methpat can summarise and visualise epiallelic DNA methylation results from targeted amplicon, massively parallel sequencing of bisulfite converted DNA in a compact and interpretable format. Unlike currently available tools, Methpat can visualise the diversity of epiallelic DNA methylation patterns in a sample.

**Electronic supplementary material:**

The online version of this article (doi:10.1186/s12859-016-0950-8) contains supplementary material, which is available to authorized users.

## Background

In mammals, the predominant and most widely studied DNA methylation mark occurs at CpG dinucleotide (CpG) palindromic sequences [1]. The vast majority of methods that investigate DNA methylation utilise bisulfite treatment of genomic DNA followed by PCR amplification to distinguish methylated from unmethylated CpG sites [2–5]. Bisulfite treatment discriminates methylated from unmethylated cytosines by selectively reacting with unmethylated cytosines to generate uracil. During the subsequent first step of PCR amplification, the uracils are read as thymine. Conversely, methylated cytosines do not react with the bisulfite reagent and remain as cytosines after PCR amplification [6]. DNA methylation readouts at single sites employing bisulfite conversion become analogous to genotyping assays by detecting either a cytosine or thymidine at the C position of a CpG site and are interpreted as methylated or unmethylated cytosines respectively.

An epiallele refers to a distinct pattern of methylation, typically over a short genomic region [7, 8]. In addition to the methylation state given for each CpG site, the pattern of DNA methylation of all CpG sites across the epiallelic or clonal template can also be characterised [7]. Indeed, in terms of biological function, CpG methylation should be often considered in an allelic fashion over multiple adjacent CpG sites [9, 10].

However, currently most studies summarise data into average percentage values at each CpG site thus losing the positional pattern information of DNA methylation across each clonal template [9]. Analysis platforms such as the Illumina Infinium BeadArray [11], bisulfite pyrosequencing [12] and SEQUENOM™ EpiTYPER™ [13] use bisulfite mediated chemistry to discriminate the methylation state of CpG sites but summarise measurements into percentage values across each CpG site or region of interest. Percentage methylation described in most DNA methylation studies hides important pattern and positional information of DNA methylation with potential functional and regulatory relevance [7]. It is only with clonal sequencing approaches [1, 14, 15], whole genome bisulfite sequencing [16] or reduced representation bisulfite sequencing [17], that the methylation state of individual CpG sites within a genomic DNA template can be readily measured in a digital sense, as methylated or not, allele by allele.

Imprinted regions of the genome such as *IGF2/H19* and *MEST* typically display two epialleles, where one is completely methylated and the other is unmethylated. The loss of imprinting at such loci leads to syndromic complications [18, 19]. Average DNA methylation across these loci are typically presented as 50 % methylation but the pattern of DNA methylation at each epiallele is lost [7].

Heterogeneous DNA methylation describes the phenomenon where different contiguous CpG sites have different levels of methylation. DNA methylation heterogeneity can arise in a variety of ways including but not limited to: (i) more than a single population of cells is analysed that differ in DNA methylation at the locus of interest, (ii) the locus of interest is imprinted i.e. two different epialleles are present in each cell or, (iii) the locus is inherently heterogeneous in its DNA methylation composition. It is only using clonal sequencing approaches with allelic outputs, high resolution melting (HRM) [7, 20], or a novel ligation mediated approach [10] that heterogeneous DNA methylation can be detected. It is also inferred by varying methylation at CpG sites e.g. from Pyrosequencing. Importantly, the number of methylated alleles can be substantially underestimated unless clonal approaches are used [20]. Clonal sequencing is currently the best method to investigate heterogeneous DNA methylation and the extent of epiallelic methylation patterns that exist within a single sample [15].

Until recently, it has been cost prohibitive to assess the complexity of methylation patterns, as large number of clones need to be individually sequenced to determine the extent of heterogeneous DNA methylation. As one clone represents a single epiallele, many tens to hundreds of clones need to be sequenced to gain a true representation of different epialleles in a sample. The introduction of massively parallel sequencing enables the sequencing of many thousands of DNA templates from multiple regions simultaneously providing a true representation of the diversity and extent of heterogeneous DNA methylation patterns derived from a given sample. However, as the number of clones sequenced increases, the ability to analyse and present this type of data then becomes a significant challenge, and at this time, there are very few software tools available to manage such data from massively parallel sequencing experiments [21, 22]. Some visualisation and analysis tools are available for Bisulfite Sanger Sequencing including BiQ Analyzer [23], MethVisual [24], QUMA [25], BISMA [26]. However, these tools do not scale up with massively parallel sequencing having been designed for Sanger sequencing. BiQ Analyser HiMod is a tool that enables visualisation of high throughput sequencing of 5-methylcytosine and other methyl-variant modifications [27] however, results are expressed in percentage methylation values masking allelic methylation patterns.

In this study, we have developed Methpat, a software tool which processes bisulfite sequencing data following Bismark alignment [28] and summarises DNA methylation according to epiallelic methylation patterns. This software has been used to analyse multiplex bisulfite amplicon PCR coupled to massively parallel deep sequencing on a range of primary haematopoietic tissue samples and model cancer cell lines to observe the extent of heterogeneous DNA methylation. Methpat is also able to create publication-ready, compact visualisations of the summarised data showing heterogeneous DNA methylation patterns in a space efficient and comprehensible manner.

## Materials, methods and implementation

Samples, library preparation, sequencing and sequence alignment. Details of sample preparation, library generation, sequencing and sequence alignment protocol employed are summarised in the Additional file 1. Human samples used in this study were approved for research by The Royal Children’s Hospital Human Research Ethics Committee (RCH HREC#27138E).

### Methpat—a tool to summarise epiallelic DNA methylation patterns

We have developed the software tool, Methpat to summarise and visualise the resultant epiallelic DNA methylation patterns from multiplex bisulfite amplicon experiments. Source code is available on GitHub (http://bjpop.github.io/methpat/). Methpat takes the output from bismark_methylation_extractor and summarises the methylation state of each CpG site within each amplicon template sequenced. DNA methylation patterns are then counted and their abundance is summarised into a tab delimited text file amenable for further downstream statistical analyses. Methpat also outputs a standalone HTML file that provides a visualisation of the DNA methylation pattern of each amplicon of interest and a visual summary of their abundance in each sample. A range of visualisation settings are customisable so that the end-user can change the settings to facilitate interpretation of the data and generate publication-ready figures. These options include presenting pattern counts as a percentage of the total, as absolute count or log-scaled counts (Additional file 2: Figure S1). Patterns can be arranged in order either by count abundance or by DNA methylation state. Colours within the visualisation can also be modified (Additional file 3: Figure S2), and the image saved as a PNG file for presentation or publication.

## Results

### Bismark alignment of sequencing data and statistics

After evaluating a range of bisulfite-aware massively parallel alignment software [29], we decided to use Bismark [28] with the highest mapping efficiency and highest proportion of concordantly mapped reads across the aligners compared to unique alignments in our previous study [29]. In addition, Bismark produces an output string that enables the processing of epiallelic DNA methylation patterns when parsed. , We developed Methpat to read this output and summarise the data in a compact and interpretable manner.

Using the stringent criterion of no mismatches within the initial 28 nt seed sequence during alignment and discarding non-unique alignments, the range of unique read alignments among the samples analysed ranged from 3,691 to 275,040 reads in total, corresponding to a mapping efficiency ranging from 7.9 to 55.3 % (Table 1). The total number of cytosine residues analysed within each sample ranged from 151,722 to 11,313,285 and includes CpG dinucleotide and non-CpG cytosine residues (Table 1). An indirect measure of bisulfite conversion efficiency was calculated by determining the percentage methylation at CHG and CHH residues in each sample. This was possible as the amplicons used in this study do not target loci where such non-CpG methylation is known to occur in humans [16] nor had human stem cells been used that are known to contain non-CpG DNA methylation [30]. CHG and CHH methylation was observed at a frequency of 0.1 to 1.0 % and 0.2 to 1.3 %, respectively, which corresponds to 98.7 to 99.9 % bisulfite conversion efficiency. This finding provides high confidence in our dataset for scoring DNA methylation states.

**Table 1:** Mapping statistics of bisulfite amplicon libraries

Sample	Mapping Efficiency	Unique Hits	Methylated CpG	Methylated CHG	Methylated CHH	Total C’s analysed
293	52.2 %	7539	64.9 %	0.2 %	0.3 %	316211
40424	55.3 %	9414	37.5 %	0.2 %	0.2 %	351086
910046	42.0 %	7060	32.6 %	0.2 %	0.3 %	299795
12a-cd19	14.9 %	48648	47.9 %	0.4 %	0.5 %	1933767
12a-cd34	30.3 %	85049	36.5 %	0.1 %	0.2 %	3703147
12a-cd45	32.4 %	109173	32.6 %	0.1 %	0.2 %	4714744
12acd33	36.2 %	161885	32.8 %	0.2 %	0.2 %	6997070
6-mda453	54.6 %	201660	84.4 %	0.8 %	1.3 %	9179816
6c-cd19	7.9 %	22258	77.8 %	0.2 %	0.3 %	777739
6c-cd33	27.9 %	20071	35.2 %	0.2 %	0.2 %	851116
6c-cd34	19.5 %	36928	49.7 %	0.2 %	0.2 %	1628107
6ccd45	33.0 %	31087	39.5 %	0.1 %	0.2 %	1314281
9a-cd19	21.2 %	39352	48.7 %	0.2 %	0.3 %	1638757
9a-cd33	31.9 %	125884	35.8 %	0.2 %	0.2 %	5459419
9a-cd34	26.2 %	77870	43.4 %	0.2 %	0.2 %	3321993
9a-cd45	46.6 %	28085	29.8 %	0.2 %	0.2 %	1211803
9awholeblood	31.5 %	97532	30.8 %	0.2 %	0.2 %	4081834
brl	49.3 %	9107	32.7 %	0.2 %	0.4 %	398977
caco	19.6 %	129536	78.1 %	0.2 %	0.2 %	4512574
dg75	51.7 %	10827	57.2 %	0.3 %	0.3 %	489096
ekvx	23.0 %	115915	63.1 %	0.2 %	0.2 %	4494359
hela	43.1 %	41650	55.9 %	0.2 %	0.2 %	1731811
hepg2	39.2 %	24667	63.4 %	0.3 %	0.3 %	971693
ht1080	40.7 %	4586	67.0 %	0.2 %	0.4 %	176188
htb22-col	30.9 %	45576	79.9 %	0.2 %	0.2 %	1863098
jwl	31.3 %	18814	42.7 %	0.2 %	0.2 %	771188
k562	49.7 %	144791	55.9 %	0.3 %	0.3 %	6230391
ls174t	41.2 %	3691	57.2 %	0.2 %	0.3 %	151722
mcf7	30.0 %	87404	71.6 %	0.8 %	0.8 %	3786412
mda-mb231-bag	29.0 %	94811	77.3 %	1.0 %	1.1 %	4171147
nalm6	43.6 %	37669	85.8 %	0.2 %	0.2 %	1569041
nccit	44.0 %	31656	45.7 %	0.4 %	0.3 %	1406165
ovcar8	32.3 %	46864	63.4 %	0.3 %	0.3 %	1917527
sknas	21.6 %	275040	27.7 %	0.1 %	0.2 %	11313285
u231	14.0 %	123302	74.8 %	0.4 %	0.2 %	4389352

Furthermore, two amplicons targeting unique regions within the human genome that contain no CpG sites were used to determine the bisulfite conversion efficiency in an orthogonal manner. Of the reads that passed alignment criteria for a subset of samples, we found that all non-CpG cytosines were converted in our experiment (Additional file 4: Figure S3). Mapping efficiency is one of many metrics used to determine the quality of the data and would suggest data from 6c-cd19 was not nominal. However, across all samples analysed, the bisulfite conversion efficiency was very high and was therefore included for visualisation using Methpat.

For the target regions analysed, an overall DNA methylation level ranging from 27.7 to 85.8 % was observed. In the lower ranges, the samples were mainly primary human tissue and non-cancerous cell lines while many model cancer cell lines demonstrated higher overall DNA methylation levels. This observation was expected, given that the amplicons selected for analysis were predominantly from promoter regions of genes known to be hypermethylated in cancer (Additional file 5: Table S2).

### Methpat analysis of DNA methylation demonstrates a wide diversity of DNA methylation patterns

#### DNA methylation of FOXP3 in primary haematopoietic cells

**Fig. 1:**
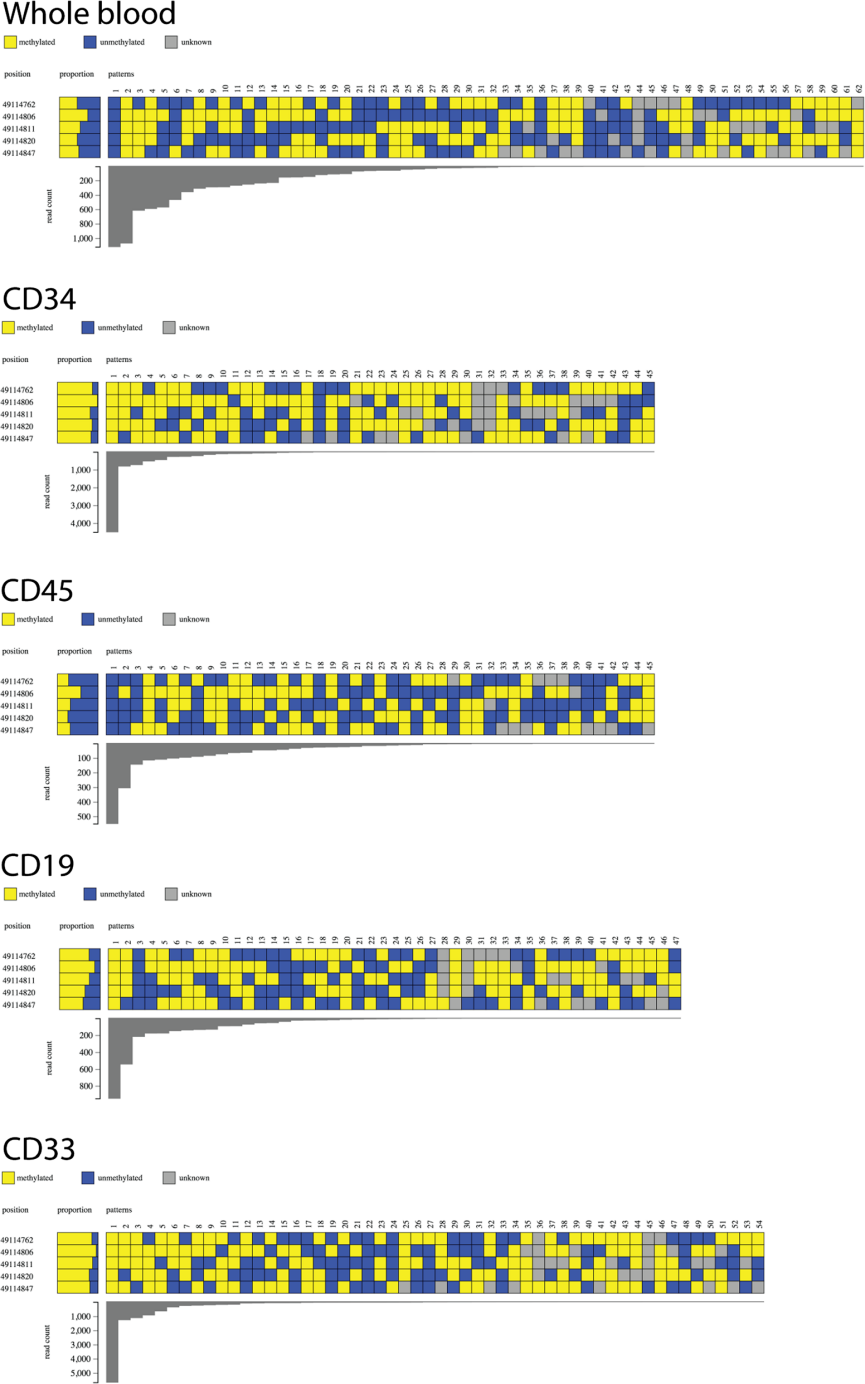
Methpat visualisation of DNA methylation at the *FOXP3* gene promoter region. Samples from one individual (blood) fluorescence activated cell sorted (FACS) into various haematopoetic compartments were assessed for DNA methylation and analysed by Methpat. DNA methylation across this locus varies according to cell type. Furthermore, the diversity of epialleles within each cell type analysed also varies with one or two patterns dominating the read counts

The promoter region of *FOXP3* was analysed for DNA methylation to validate the amplicon next generation sequencing, bioinformatics analysis and Methpat visualisation pipeline. Amplicons obtained from whole blood and subpopulations of cells from bone marrow were analysed from a single individual, from which, a diverse range of DNA methylation states and their abundance was observed. Analysis of whole blood showed that although the majority of epialleles were either completely methylated or completely unmethylated at CpG sites (Fig. 1), there were a diverse array of methylation patterns present (62 in total). This could reflect the cellular composition of whole blood, such that a number of cell types exist with a variable DNA methylation state at *FOXP3*. In contrast, DNA extracted from CD34, CD19 and CD33 positive subpopulations were found to be largely methylated at *FOXP3.* The CD45 positive compartment was unmethylated (Fig. 1). This was in line with previous investigations on similar sample types [31].

#### Methpat can visualise imprinted loci

**Fig. 2:**
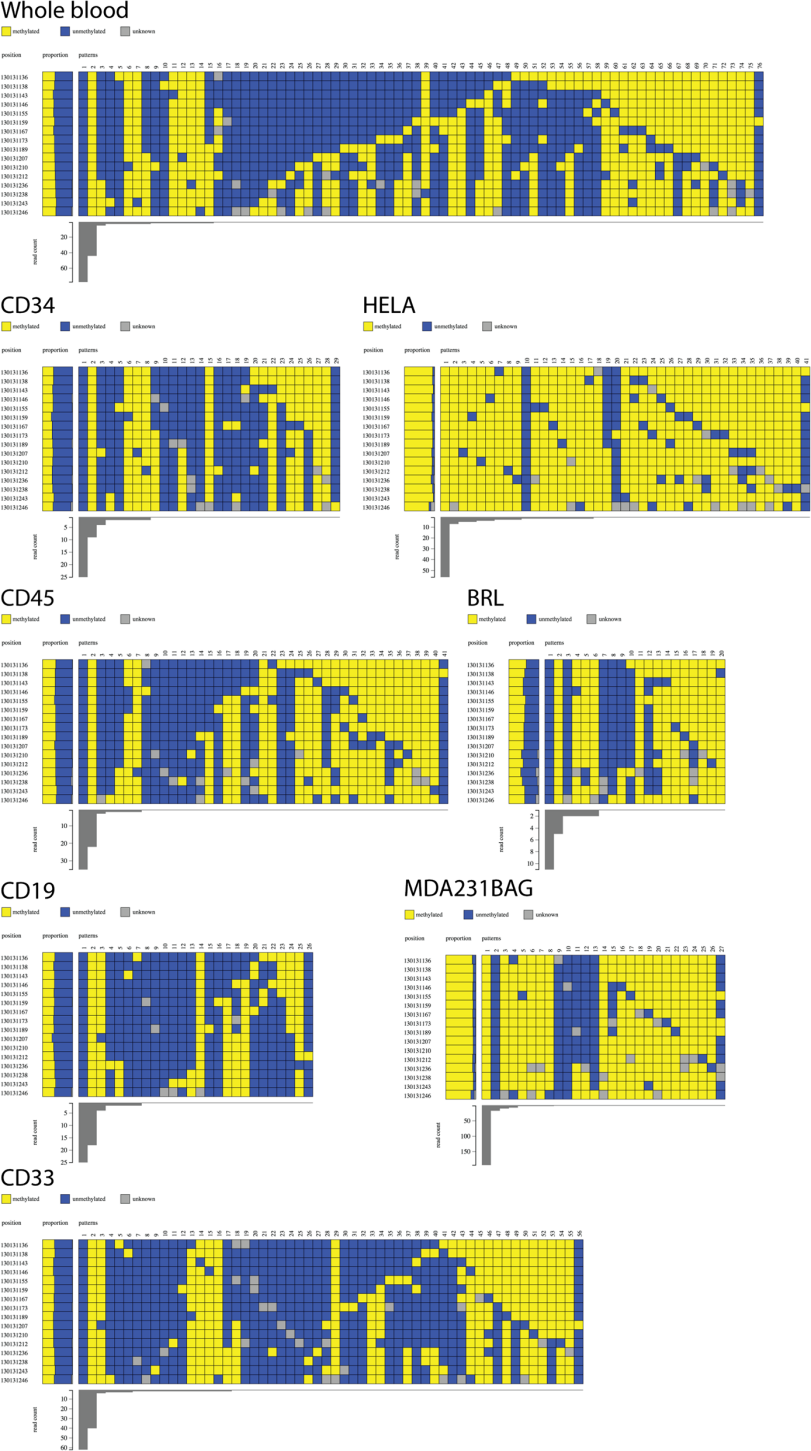
Methpat visualisation of DNA methylation at the *MEST* imprinted region on a range of primary cells (CD34, CD45, CD19 and CD33) and tissue (Whole blood), model cancer cell lines (HeLA and MDA-MB-231-BAG) and a normal lymphoblast cell line (BRL). The methylation status of *MEST*, expected to be ~50 % was observed in all normal sample types. The cancer cell lines demonstrate methylated *MEST*. In addition, Methpat visualizes the epiallelic diversity of *MEST* in all these samples

The extent of DNA methylation at a known imprinted locus, *MEST,* was investigated. This locus also served as a PCR amplification bias control as the DNA methylation state was expected to be 50 %, as this locus is comprised of two populations of epialleles where one is completely methylated while the other is completely unmethylated. Both epialleles were clearly identified in whole blood, CD34, CD33, CD19 and CD45 positive samples (Fig. 2) with the unmethylated epiallele more abundant than the methylated epiallele. Additional epialleles of varying DNA methylation patterns were also identified but at a significantly lower abundance (Fig. 2). The same imprinted state was also observed in the lymphoblastoid cell line, BRL (Fig. 2). The imprinting of *MEST* is known to be disrupted in model cancer cell lines [32]; HeLa and MDA-MB-231-BAG cell lines were observed to have predominantly hypermethylated epialleles at this locus (Fig. 2) and is in keeping with publically available datasets with these cell lines found on ENCODE [33].

#### Methpat visualisation of gene promoters associated with cancer

**Fig. 3:**
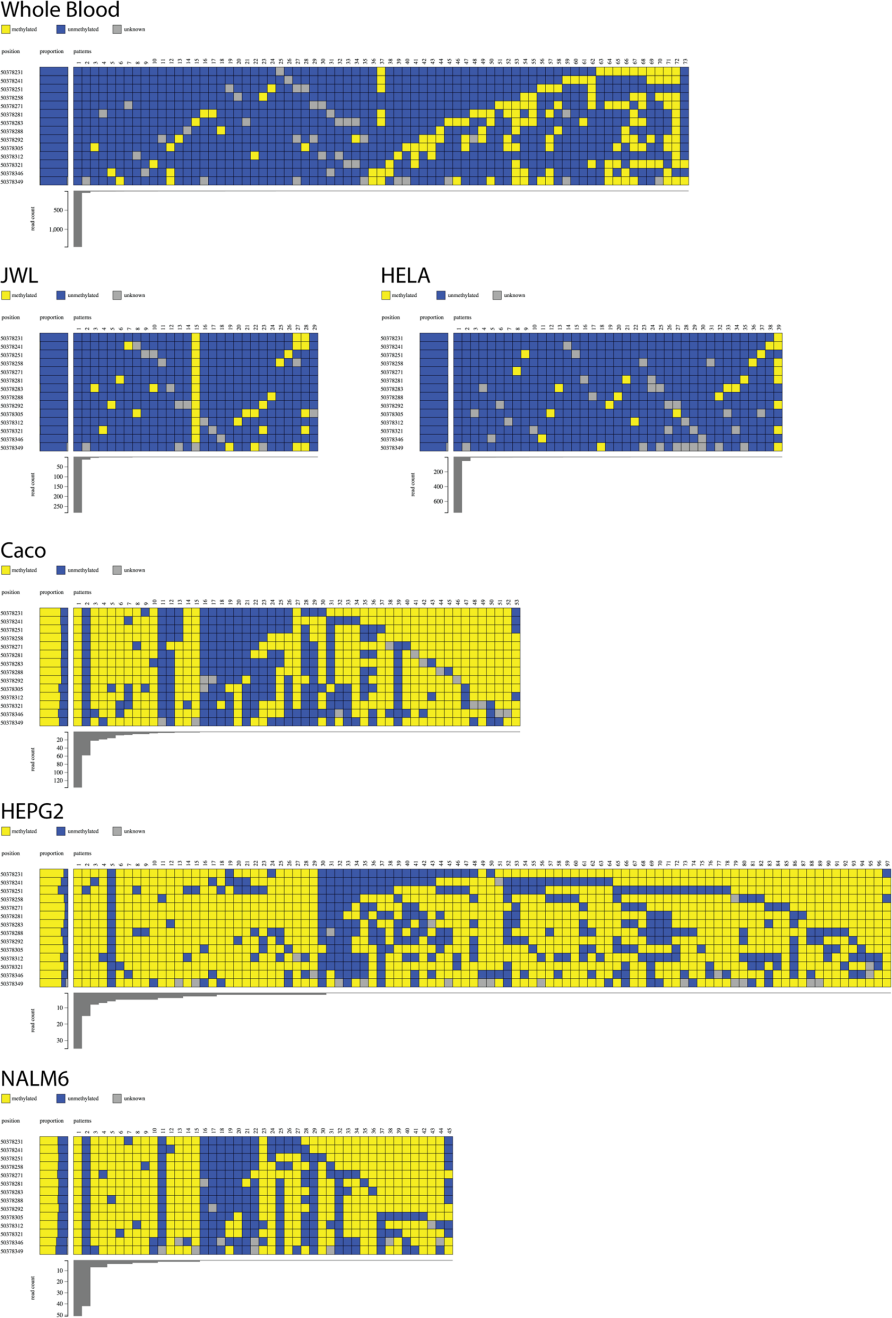
Methpat visualisation of DNA methylation at the *RASSF1A* gene promoter region. Methylation of *RASSF1A* is present in cancer cell lines (Caco, HEPG2 and NALM6) with the exception of HeLa. Examples of *RASSF1A* methylation in whole blood and a normal lymphoblast cell line (JWL) are also shown

The methylation state of the *RASSF1A* gene promoter, which is known to be methylated in cancer [34, 35], was determined. In wild-type whole blood and the lymphoblast cell line JWL, unmethylated epialleles were primarily observed with a significant number of other much lower abundance epiallele states with varying patterns of DNA methylation (Fig. 3). HeLa was also unmethylated at *RASSF1A* while other cancer cell lines, HEPG2, NALM6, Caco (Fig. 3), MCF7 and NCCIT (Additional file 6: Figure S4) were predominantly hypermethylated. Of note, the diversity and range of the DNA methylation state of epialleles are much greater than might be expected of cell lines.

**Fig. 4:**
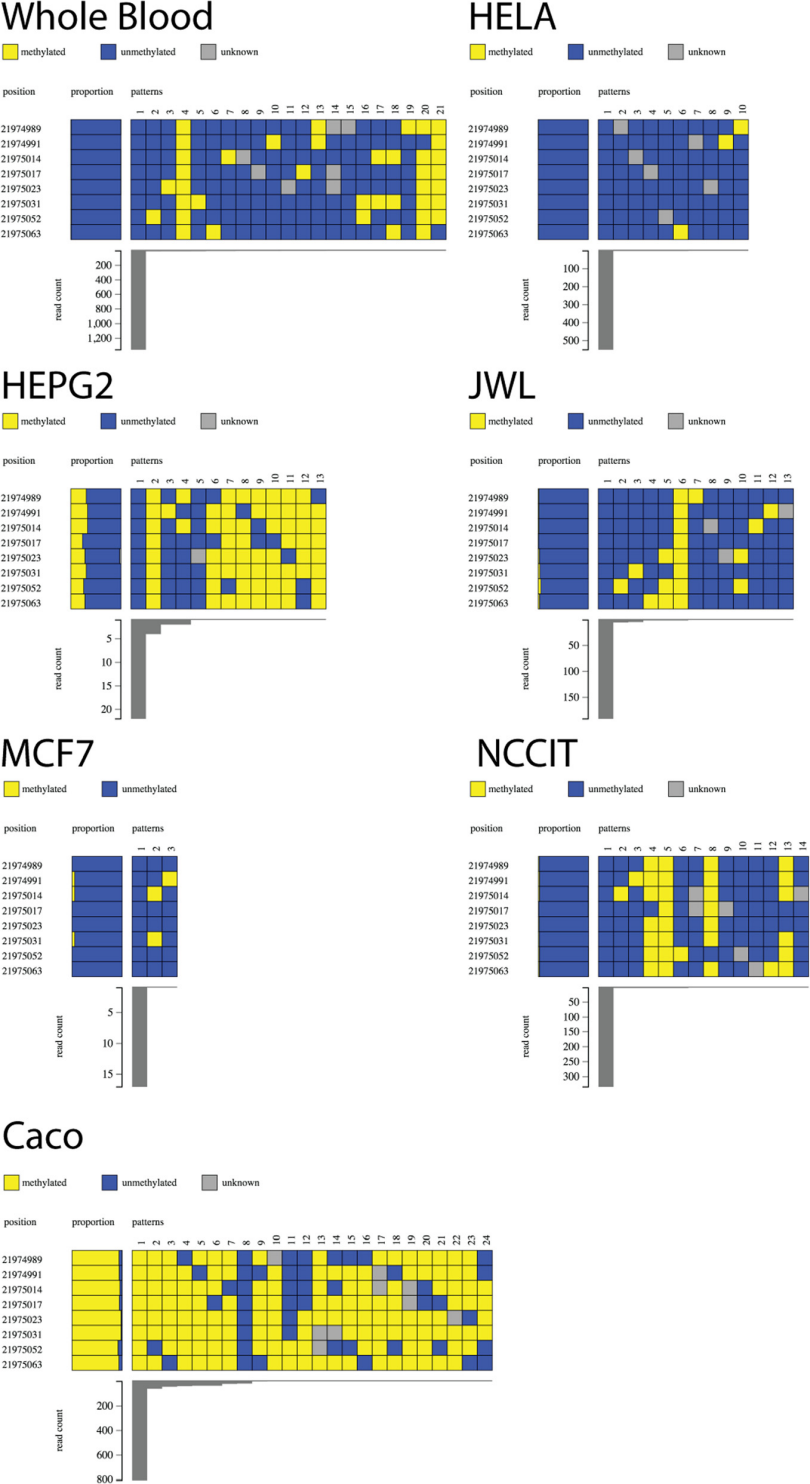
Methpat visualisation of DNA methylation at the *CDKN2A* gene promoter region

We also investigated DNA methylation of the gene promoter of *CDKN2A*, at which DNA methylation is also seen in many cancers [36] (Fig. 4). We found that the unmethylated epiallele was most abundant in normal whole blood, HeLa, HEPG2, JWL, MCF7 and NCCIT. In contrast, Caco was hypermethylated at this locus. Interestingly, in wildtype whole blood and the cell lines HEPG2, JWL, and NCCIT, the completely methylated epiallele could be observed but was at very low abundance compared to the unmethylated epiallele (Fig. 4). We confirmed that these alleles did not arise from incomplete bisulfite conversion artefacts as all non-CpG cytosines were converted to thymidine.

#### Methpat visualisation of mitochondrial genome DNA methylation

**Fig. 5:**
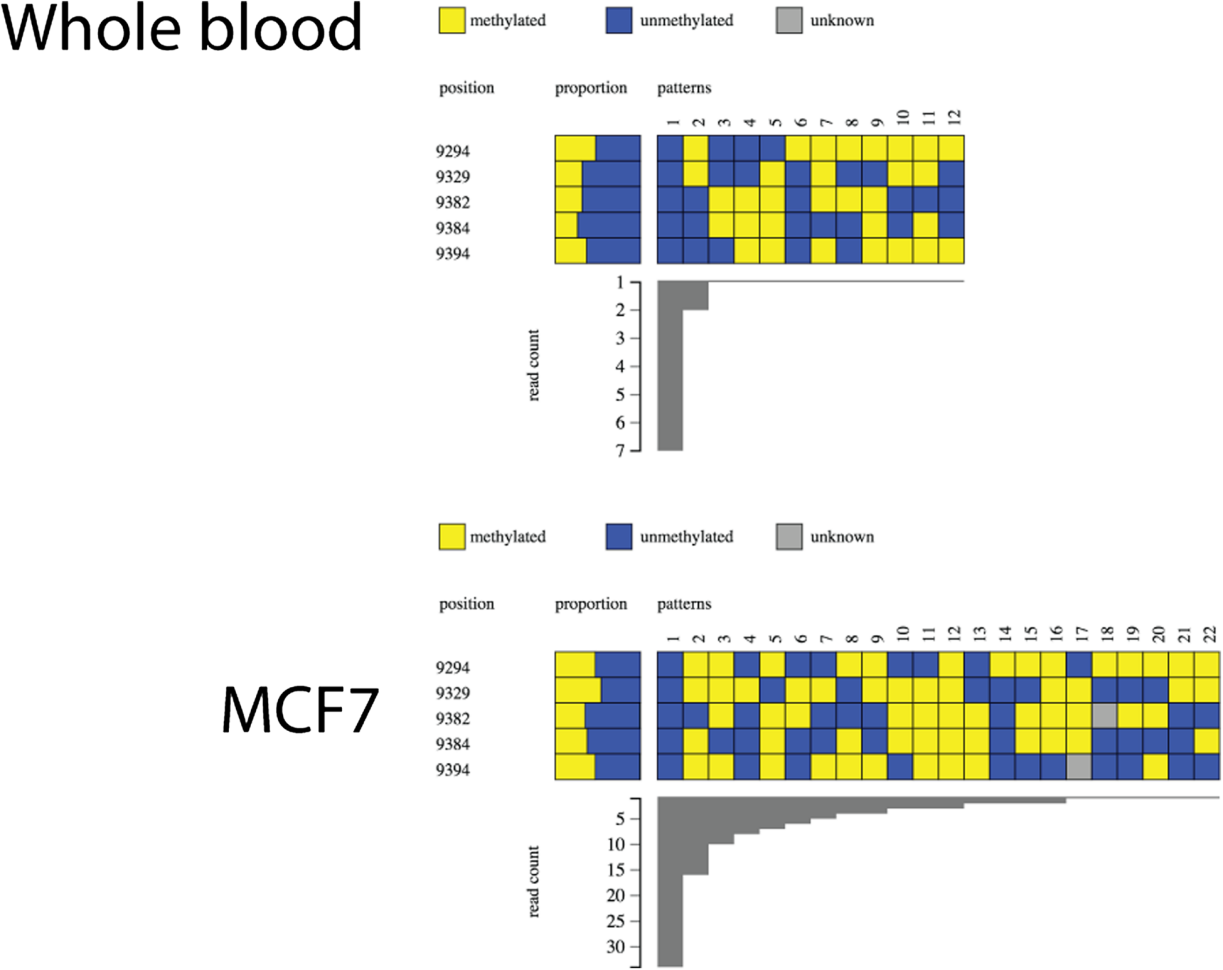
Methpat visualisation of DNA methylation within the D-Loop regulatory region of the mitochondrial genome

Bisulfite amplicon primers to the mitochondrial DNA D-loop regulatory sequence were included in the analysis to determine the DNA methylation state of the mitochondrial genome. The predominant epiallele was found to be unmethylated across most samples analysed; however, there was a significant range in the abundance of epialleles with variable DNA methylation state across all samples (Fig. 5, Additional file 7: Figure S5), suggesting that DNA methylation of the mitochondrial genome was present [37] but appeared to be independent of the disease status of the sample. This is in keeping with recent observations of mitochondrial genomic DNA methylation in human cells [38, 39]. We again confirmed that these alleles did not arise from incomplete bisulfite conversion artefacts as all non-CpG cytosines were converted to thymidine.

## Discussion

Most studies investigating DNA methylation using conventional sequencing approaches represent DNA methylation into percentage values at each CpG site and in turn, do not show important positional information encoded within the epiallelic DNA methylation patterns. A comparison of features between methylation visualisation tools is summarised in Table 2. We have developed a new software tool called Methpat that processes output files from Bismark to visualise DNA methylation sequencing data by epialleles. Methpat facilitates visualisation of high throughput sequencing data after Bismark analysis and does not attempt to determine the success of a particular experiment. This is left to the investigator to interpret the metrics from Bismark prior to Methpat visualisation. We demonstrate the utility of Methpat by examining the DNA methylation pattern abundance and epiallelic DNA methylation states that are lost when DNA methylation is summarised as percentage DNA methylation.

**Table 2:** Alternative DNA methylation Analysis and Visualisation Tools

Software	Program Language and Implementation	Analysis Process	Visual Output	Input file	Output file	Epiallelic Counts	Experiment Quality Check
Methpat	Python, pip install, URL available to install files locally	Summarises Bismark output	Interactive HTML and summary text file of epiallele counts. Scalable PNG file	Bismark methylation extractor output, user-defined BED format file	HTML and tab delimited text file	Yes	No, leverages Bismark
Bismark	command line,Python, requires bwa	Performs alignment to bisulfite reference genome	None, generates BAM files for visualisation with SeqMonk or IGV	fastq file	BAM and tab deliminted text files	No	Yes calculates C to T conversion
BSPAT	Java/JSP web interface	Visualisation and summarisation of Bismark output	PNG file and UCSC Genome Browser file	Bismark output, fastq files	Text file summary, PNG and UCSC Genome Browser BED file	Yes	No
MPFE	R library, Bioconductor	Calculates probabilities that epialleles are true	R image outputs	Table of read counts from bisulfite sequencing data	Derived statistics and plots	Yes	Yes
Methylation plotter	R library, shiny interactive web application	Visualises beta DNA methylation values	Interactive webpage with setting options to adjust a static image of DNA methylation values for each sample. PNG and PDF output.	Text file containing matrix of sample vs beta value at each CpG of interest	PDF and PNG image file	No	No
RnBeads	R library, Bioconductor	Processes summary data from other software for visualisation	Interactive HTML and UCSC Genome browser track hub files. PNG files	BED file	HTML summary	No	Yes
coMET	R library, Webserver for analysis	For EWAS studies. Analyses derived matrix files	Image files of plots with genomic locations.	Text matrix files	Image files	No	No

*EWAS* epigenome-wide association studies using Illumina Infinium HM450 BeadArrays

Methpat operates on Bismark output files and further summarizes this data into an interactive visualization that can be quickly interpreted within a web-browser. It can be executed locally to generate an HTML file which can be hosted remotely through the Internet or visualized locally on the most common web browsers (Chrome, Safari, Firefox, Internet Explorer). This feature which is unique to Methpat, is a major advantage. At this stage, Methpat does not have capability as a “genome-browser” to look at DNA methylation patterns at a genome-scale because it was designed for targeted deep sequencing of amplicons, however, we have made the source code available for further development by the research community to further improve Methpat (http://bjpop.github.io/methpat/).

We demonstrated the importance of calculating epiallelic abundance on the imprinted locus *MEST,* where we showed two predominant populations of epiallelic DNA methylation patterns, one completely methylated and the other completely unmethylated. Such patterns cannot be interpreted with percentage values at each CpG site as heterogeneous DNA methylation or, a sample containing a heterogeneous population of cells with variable DNA methylation states could give rise to the same percentage value [7]. Using Methpat to visualise the diversity of epialleles enables the inference at least of the existence of heterogeneous DNA methylation, or, the detection of heterogeneous populations of cells as demonstrated by investigating *FOXP3* in whole blood and subpopulations of the haematopoietic compartment.

Of interest, in some model cancer cell lines, we observed a wide and diverse range of methylated epialleles. Having ruled out to the best of our ability any bisulfite conversion or PCR amplification artefacts, our results suggest that even within apparently homogeneous cell lines, the methylation state at a subset of gene promoters analysed is heterogeneous. This could be due to the nature of cell culture where the phenomenon of increasing DNA methylation is observed with increasing passage [40, 41], plasticity, or the setting of epigenetic memory of a sub-population of cells in the culture [42]. The detection of completely methylated epialleles of the *CDKN2A* gene promoter in whole blood and in other samples interrogated supports the validity of our approach, and indicates that Methpat provides a new tool to enable the detection of low level DNA methylation [43, 44]. The functional and biological implications of our current findings remain unclear, however, further investigation with appropriate specimens using Methpat is warranted.

We investigated mitochondrial DNA methylation and believe our analysis is one of the first accounts of characterising epiallelic DNA methylation within the D-loop regulatory region of the mitochondrial genome. Our study confirms observations of DNA methylation within the mitochondria [37–39]. Given there can be many thousands of copies of the mitochondrial genome per cell, it is not possible at this stage to determine the providence of the methylation states we have identified. The issue of heteroplasmy for mutations in the mitochondrial genome [45] apply for DNA methylation and techniques to address heteroplasmy could be applied to investigate DNA methylation within the mitochondrial genome further [46]. By visualising DNA methylation patterns within the mitochondrial genome, Methpat can facilitate insight towards new biomarkers of disease [47].

While our current strategy and experimental results are unable to resolve PCR amplification artefacts (over-representation of particular sequence reads because of amplification), incorporation of unique molecular identifiers [48] could resolve this in future studies.

## Conclusions

In summary, we demonstrate the feasibility of multiplex bisulfite amplicon deep sequencing to identify the extent of DNA methylation epialleles in a range of human samples. We have developed a software tool, called Methpat, which enables the summarisation and visualisation of DNA methylation sequencing data in the context of epiallelic information.

### Availability of data and materials

The raw amplicon sequencing data, Bismark alignments and Methpat output files associated with this manuscript have been published with the DOI 10.1186/s13742-015-0098-x.

Methpat software can be obtained from this URL. (http://bjpop.github.io/methpat/)

## Additional files

Additional file 1:
**Sample preparation, library preparation and sequencing methods.** (DOCX 132 kb) Additional file 2: Figure S1. Example of a screenshot of Methpat visualisation. A. Epiallele representation of the patterns of DNA methylation for respective amplicon in respective sample. B. Count histogram, the abundance of each epiallele represented in A. C. Genomic co-ordinate and position of CpG of interest. D. Proportion of DNA methylation at each CpG position. E. Save button, export visualisation as a PNG file. F. Amplicon of interest. G. Legend depicting DNA methylation status. (PNG 202 kb) Additional file 3: Figure S2. Example of a screenshot of the settings page for each Methpat visualisation. A number of parameters can be changed and the visualisation replotted for ease of interpretation. (PNG 92 kb) Additional file 4: Figure S3. IGV screenshot of two amplicon regions used in this study that target DNA sequences with no CpG sites within the *RANBP17* locus. Therefore it is expected that all cytosines within this region of interest are completely converted by bisulfite treatment. This is shown here for MCF7 and MDA-MB-231-BAG. (PNG 135 kb) Additional file 5: Table S2. Bisulfite PCR primers used in this study. (XLS 15 kb) Additional file 6: Figure S4. Diverse and wide ranging epiallelic DNA methylation patterns of *RASSF1A* in MCF7 and NCCIT model cancer cell lines. (PNG 434 kb) Additional file 7: Figure S5. Epiallelic DNA methylation patterns of the D-loop regulatory region of the mitochondrial genome. (PNG 163 kb) Additional file 8: Table S1. Human Samples used in this study. (XLS 8 kb) Additional file 9: Table S3. Amplicon details required for Methpat input (hg19 coordinates). (XLS 9 kb) Additional file 10: Description of Methpat options. (DOCX 118 kb)
