# Supplementary material for: MethPat: a tool for the analysis and visualisation of complex methylation patterns obtained by massively parallel sequencing
Source: BMC Bioinformatics. 2016 Feb 24;17:98. doi: 10.1186/s12859-016-0950-8 (PMC4765133; doi:10.1186/s12859-016-0950-8)
Supplement: Additional file 1: — Sample preparation, library preparation and sequencing methods. (DOCX 132 kb) [file 12859_2016_950_MOESM1_ESM.docx]

Wong et al.

**MethPat: a tool for the analysis and visualisation of complex methylation patterns obtained by massively parallel sequencing.**

**Supplementary Methods.**

Samples, library preparation and sequencing. Genomic DNA from primary haematopoietic tissues and model cell lines was extracted using a standard phenol-chloroform and ethanol precipitation protocol. A table of samples analysed and a brief description of each is summarised in Supplementary Table 1. Primary human tissue samples were collected with appropriate consents under Human Research Ethics Committee approval at The Royal Children’s Hospital (Melbourne), HREC27138. Bisulfite conversion of genomic DNA (2µg) was performed using a MethylEasy Xceed v2.0 Bisulfite Modification Kit according to the manufacturer’s instructions (Human Genetic Signatures, Sydney, Australia). Multiplex bisulfite PCR was performed using the Multiplex PCR Master Mix (Qiagen, Hilden, Germany) with a combined multiplex primer pool concentration of 500nM in a final PCR reaction volume of 20µL. A list of primer sequences is found in Supplementary Table 2. PCR cycling conditions included an activation step of 15 minutes at 95°C followed by 10 cycles of denaturation at 95°C for 15 seconds, annealing at 58°C for 30 seconds, and extension at 72°C for 2 minutes. A further 30 cycles of denaturation at 95°C for 15 seconds, annealing at 60°C for 30 seconds and extension at 72°C for 1 minute was performed. A final extension of 72°C for 3 minutes was completed after PCR cycling. PCR amplicons were purified using AmpureXP Beads (Beckman Coulter, Sydney, Australia) according to the manufacturer’s instructions. PCR amplicons were designed to be less than 210bp (Supplementary Table 3) and size selection using AmpureXP was performed accordingly. Amplicons from each sample were then barcoded according to manufacturer’s instructions using the Illumina TruSeq Dual Indexing Kit (Illumina, San Diego, CA). Sample libraries were pooled in equimolar concentration and sequenced on an Illumina MiSeq Personal Sequencer using v3 150 bp single end chemistry according to the manufacturer’s instructions (Illumina). Raw fastq files are deposited in the Sequence Read Archive under [GSE67856](http://www.ncbi.nlm.nih.gov/geo/query/acc.cgi?token=qruhwasexjgtbmh&acc=GSE67856). (Reviewer link included here, http://www.ncbi.nlm.nih.gov/geo/query/acc.cgi?token=qruhwasexjgtbmh&acc=GSE67856).

Sequence Alignment. Raw fastq files were first checked for read quality using fastQC (<http://www.bioinformatics.babraham.ac.uk/projects/fastqc/>). Reads were then aligned to the hg19 reference genome assembly using the bisulfite sequencing aligner Bismark (v0.9.0) [28]. To reduce spurious alignments that arise due to sequencing errors, an alignment parameter was set that required that no mismatches were present within the initial seed sequence (set to 28nt). The bismark_methylation_extractor tool within Bismark was used to extract DNA methylation information from each read within the resultant Sequence Alignment Mapping (SAM) files.

Methpat command line options:

**usage:** methpat [-h] [--count_thresh THRESH] --amplicons AMPLICONS_FILE

[--logfile FILENAME] [--html FILENAME]

[--webassets {package,local,online}] [--title TITLE]

BISMARK_FILE

-h help option, prints the above text.

--count_thresh read count threshold, require THRESH number of reads in input file before epiallele pattern is counted.

--amplicons path to file containing BED coordinates of genomic locations labelled

--logfile set FILENAME of log output, otherwise to stdout.

--html set FILENAME of html output.

--webassets set location of D3.js libraries used for visualisation as {package, local, online}.

--title set TITLE of html file.

BISMARK_FILE path to the bismark_methylation_extractor output file for Methpat to process.
