# Supplementary figures and images for: MethPat: a tool for the analysis and visualisation of complex methylation patterns obtained by massively parallel sequencing
Source: BMC Bioinformatics. 2016 Feb 24;17:98. doi: 10.1186/s12859-016-0950-8 (PMC4765133; doi:10.1186/s12859-016-0950-8)

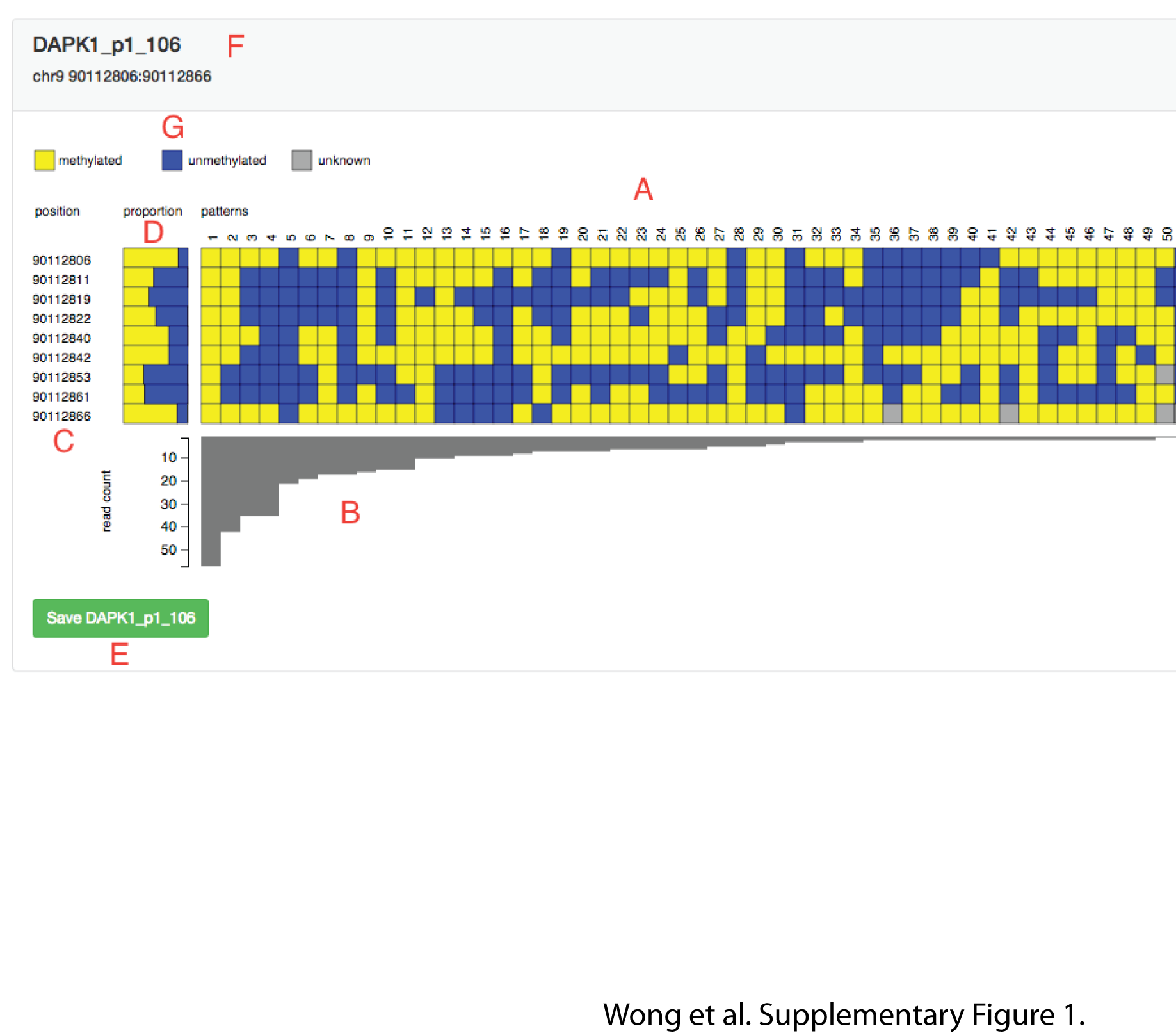

Supplement: Additional file 2: Figure S1. — Example of a screenshot of Methpat visualisation. A. Epiallele representation of the patterns of DNA methylation for respective amplicon in respective sample. B. Count histogram, the abundance of each epiallele represented in A. C. Genomic co-ordinate and position of CpG of interest. D. Proportion of DNA methylation at each CpG position. E. Save button, export visualisation as a PNG file. F. Amplicon of interest. G. Legend depicting DNA methylation status. (PNG 202 kb) [file 12859_2016_950_MOESM2_ESM.png]

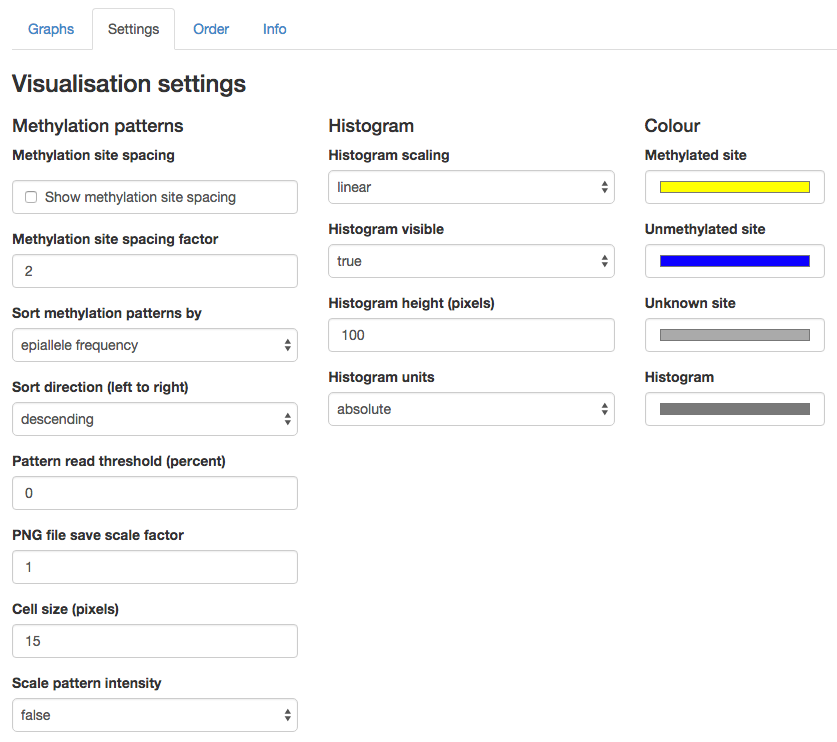

Supplement: Additional file 3: Figure S2. — Example of a screenshot of the settings page for each Methpat visualisation. A number of parameters can be changed and the visualisation replotted for ease of interpretation. (PNG 92 kb) [file 12859_2016_950_MOESM3_ESM.png]

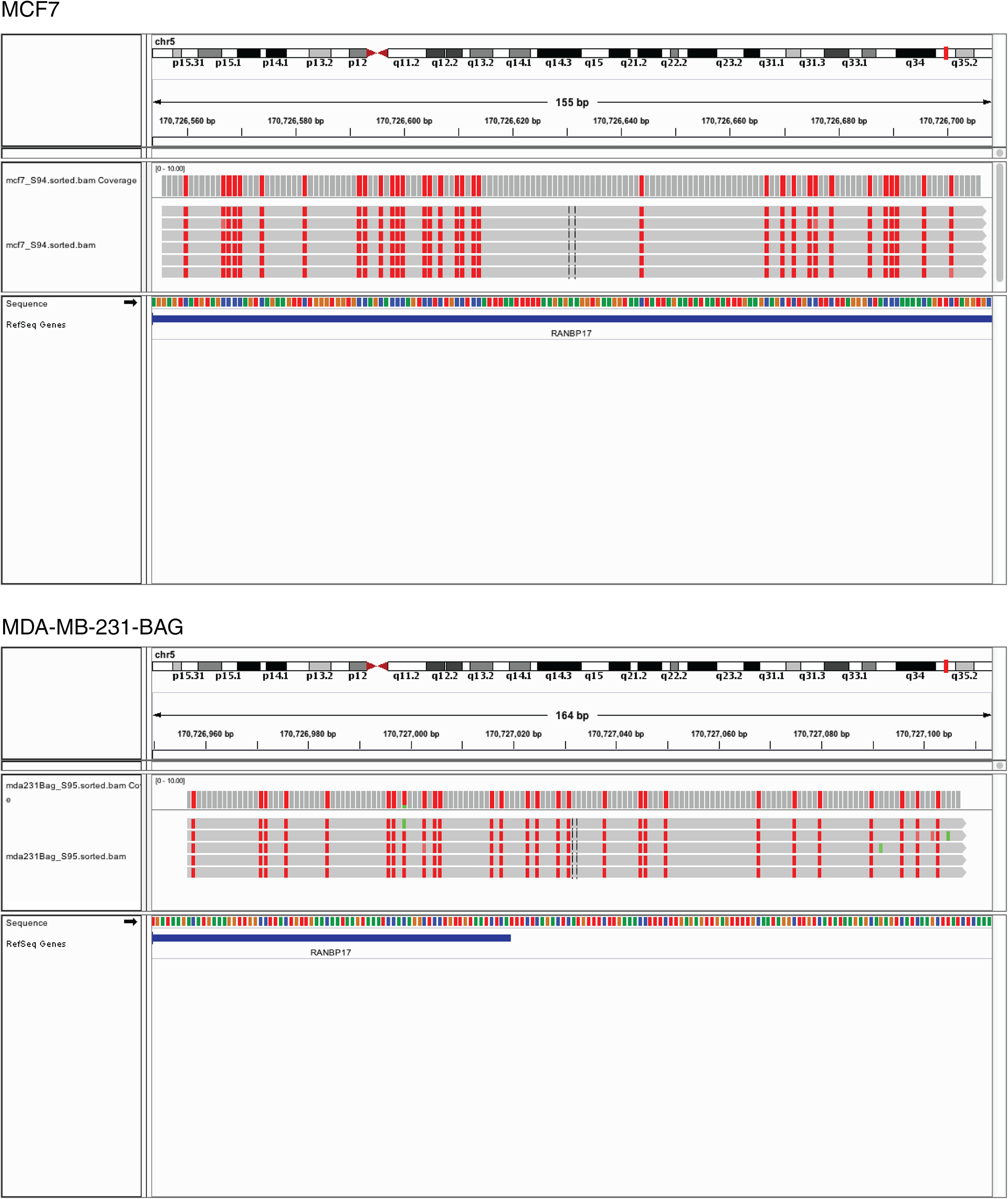

Supplement: Additional file 4: Figure S3. — IGV screenshot of two amplicon regions used in this study that target DNA sequences with no CpG sites within the RANBP17 locus. Therefore it is expected that all cytosines within this region of interest are completely converted by bisulfite treatment. This is shown here for MCF7 and MDA-MB-231-BAG. (PNG 135 kb) [file 12859_2016_950_MOESM4_ESM.png]

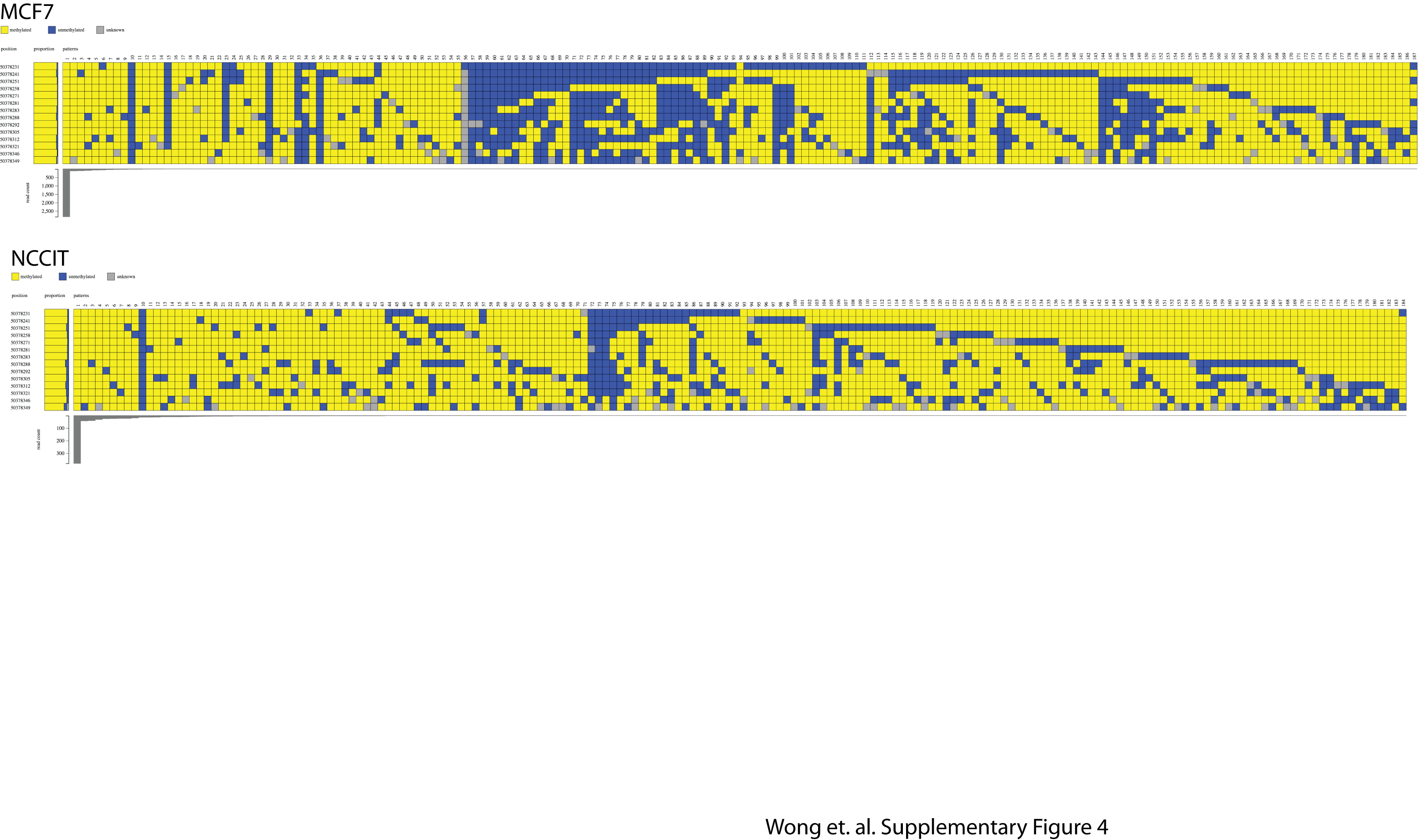

Supplement: Additional file 6: Figure S4. — Diverse and wide ranging epiallelic DNA methylation patterns of RASSF1A in MCF7 and NCCIT model cancer cell lines. (PNG 434 kb) [file 12859_2016_950_MOESM6_ESM.png]

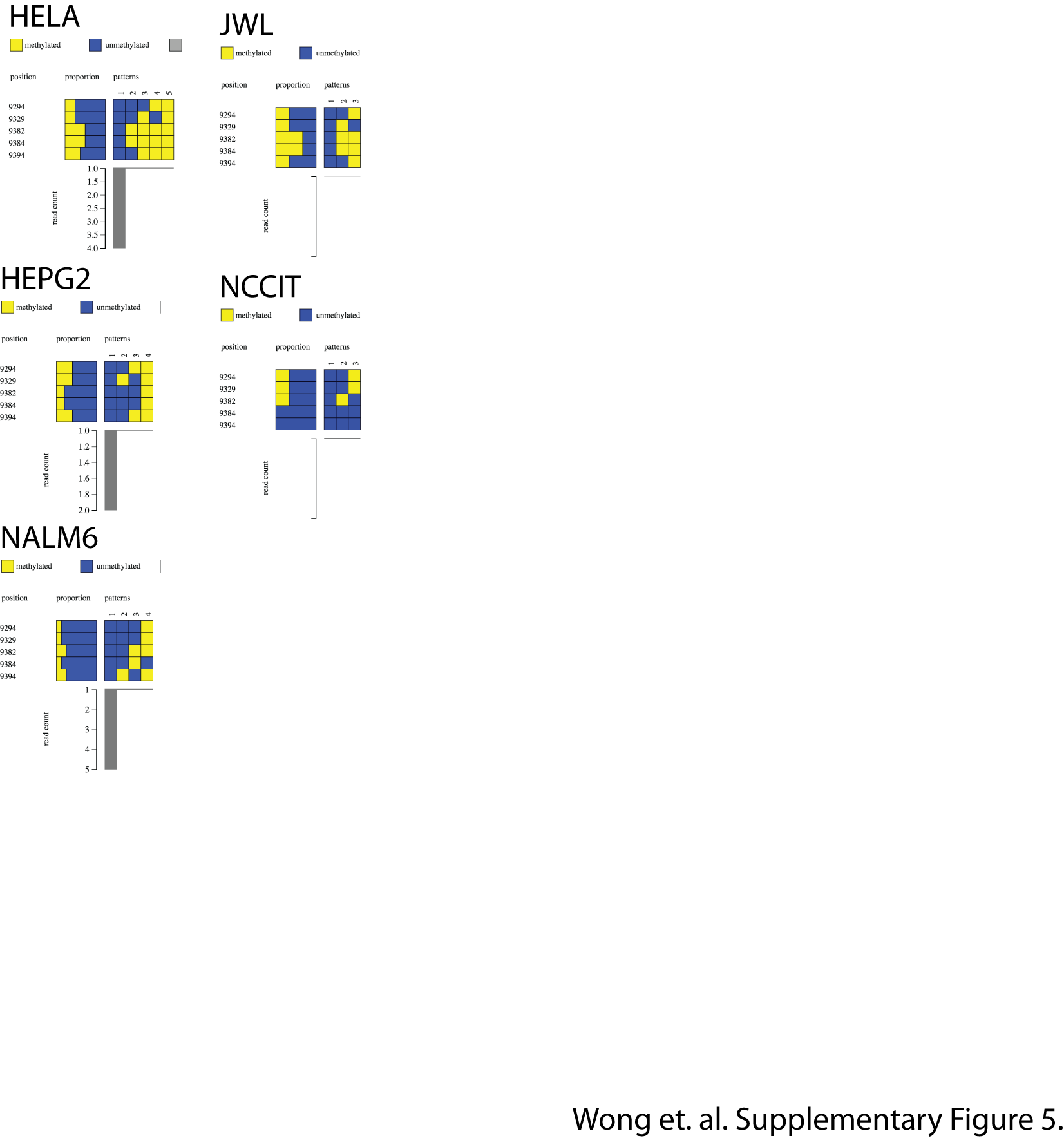

Supplement: Additional file 7: Figure S5. — Epiallelic DNA methylation patterns of the D-loop regulatory region of the mitochondrial genome. (PNG 163 kb) [file 12859_2016_950_MOESM7_ESM.png]
