# Supplementary material for: MethPat: a tool for the analysis and visualisation of complex methylation patterns obtained by massively parallel sequencing
Source: BMC Bioinformatics. 2016 Feb 24;17:98. doi: 10.1186/s12859-016-0950-8 (PMC4765133; doi:10.1186/s12859-016-0950-8)
Supplement: Additional file 10: — Description of Methpat options. (DOCX 118 kb) [file 12859_2016_950_MOESM10_ESM.docx]

**Supplementary Document. Details of the visualisation options and settings of Methpat HTML file output.**

The HTML output from Methpat can be rendered by most of the major web browsers in use including Chrome, Safari and Firefox. There are four tabs including the main graphical tab (Graphs), Settings, Order and Info tabs.

**Settings:**

- Methylation patterns:
  - Show methylation site spacing: check on this radio button to display the relative spacing between CpG sites in genomic distance (base pairs). The vertical width of the spacing is controlled by *Methylation site spacing factor*, which is a multiple of the normal (non-spaced) display of the patterns. It defaults to 2 which means that the spaced display will be twice as wide as the non-spaced display.
  - Sort methylation patterns by: The ordering of the methylation patterns across the screen (left to right). The ordering can be by "epiallele frequency" (pattern frequency count) or by "degree of methylation" (total number of methylated sites within the epiallele).
  - Sort direction (left to right): The ordering of methylation patterns across the screen in left-to-right direction. The ordering can be "ascending" or "descending".
  - Pattern read threshold (percent): only display methylation patterns with at least this percentage of reads, out of all the reads for the amplicon. This allows you to dynamically filter out patterns with low relative frequency.
  - PNG file save scale factor: the scaling factor for saving the PNG file. By default the saved PNG file will have the same size (and hence number of pixels) as the rendered graph in the web browser. You can increase this scale (and hence image size and resolution) by making this number larger. This may be useful for situations where you need a higher resolution image for the purposes of inclusion in a publication.
  - Cell size (pixels): The side-length in pixels of the coloured squares used to show the methylation patterns. Default is 15 pixels.
  - Scale pattern intensity: If set to "true" the colour intensity of each methylation pattern will be scaled to indicate its frequency. More frequent patterns are shown brighter and less frequent patterns are shown darker. This is set to "false" by default.
- Histogram:
  - Histogram scaling: How to display the vertical scale (read counts) of the histogram. The scale can be "linear" or "log".
  - Histogram visible: Should the histogram be displayed? If set to "true" then the histogram will be displayed. If set to "false" the histogram will not be displayed.
  - Histogram height (pixels): The maximum vertical height of the histogram in pixels. Defaults to 100 pixels.
  - Histogram units: The units of the vertical scale on the histogram. The units can be "absolute" or "relative". The absolute scale shows raw counts. The relative scale shows the percentage of all reads (total reads) for each amplicon.
- Colour:
  - Methylated site: The colour to use for a methylated site.
  - Unmethylated site: The colour to use for an unmethylated site.
  - Unknown site: The colour to use for a CpG site with unknown methylation state.
  - Histogram: The colour of the bars in the histogram.

**Order:**

Within the order tab it is now possible to arrange the order of each amplicon displayed from top to bottom.
